# Development and assessment of novel virtual COVID-19 trainer-of trainers course implemented by an academic–humanitarian partnership

**DOI:** 10.1080/16549716.2021.2010391

**Published:** 2022-01-10

**Authors:** Ramu Kharel, Janette Baird, Himanshu Vaishnav, Nidhi Chillara, J. Austin Lee, Alicia Genisca, Alison Hayward, Vlatko Uzevski, Asmaa Elbenni, Adam C. Levine, Adam R. Aluisio

**Affiliations:** a Division of Global Emergency Medicine, Warren Alpert Medical School, Brown University, Providence; b Department of Emergency Medicine and Pediatrics, Warren Alpert Medical School, Brown University, Providence; c Department of Emergency Medicine, Brown University, Providence, RI, USA; d Medical School of International Health, Ben Gurion University of the Negev, Beersheba, Israel; e Department of Emergency Medicine, Warren Alpert Medical School, Brown University, Providence; f Project HOPE, Millwood, VA, USA

**Keywords:** COVID-19, education, public health, virtual education, global health

## Abstract

**Background:**

In response to the coronavirus disease (COVID-19) pandemic, Project HOPE®, an international humanitarian organization, partnered with Brown University to develop and deploy a virtual training-of-trainers (TOT) program to provide practical knowledge to healthcare stakeholders. This study is designed to evaluate this TOT program.

**Objective:**

The goal of this study is to assess the effectiveness of this educational intervention in enhancing knowledge on COVID-19 concepts and to present relative change in score of each competency domains of the training.

**Methods:**

The training was created by interdisciplinary faculty from Brown University and delivered virtually. Training included eight COVID-19 specific modules on infection prevention and control, screening and triage, diagnosis and management, stabilization and resuscitation, surge capacity, surveillance, and risk communication and community education. The assessment of knowledge attainment in each of the course competency domain was conducted using 10 question pre-and post-test evaluations. Paired t-test were used to compare interval knowledge scores in the overall cohort and stratified by WHO regions. TOT dissemination data was collected from in-country partners by Project Hope.

**Results:**

Over the period of 7 months, 4,291 personnel completed the TOT training in 55 countries, including all WHO regions. Pre-test and post-test were completed by 1,198 and 706 primary training participants, respectively. The mean scores on the pre-test and post-test were 68.45% and 81.4%, respectively. The mean change in score was 11.72%, with P value <0.0005. All WHO regions had a statistically significant improvement in their score in post-test. The training was disseminated to 97,809 health workers through local secondary training.

**Conclusion:**

Innovative educational tools resulted in improvement in knowledge related to the COVID-19 pandemic, significantly increasing the average score on knowledge assessment testing. Academic – humanitarian partnerships can serve to implement and disseminate effective education rapidly across the globe.

## Background

Since late 2019, Severe Acute Respiratory Syndrome Coronavirus 2 (SARS-CoV-2)’s disease (COVID-19) quickly stressed health systems and overwhelmed resources at a local and global level. Evolving knowledge and limited training of frontline workers is a barrier to pandemic response [1]. The unprecedented global strain caused by COVID-19 has required innovative and rapid solutions to multifactorial challenges in education [2,3]. Striving to meet the demand for training on this novel illness, Project HOPE®, an international humanitarian organization that works around the world, partnered with faculty from Brown University to create and disseminate a remote, virtual training-of-trainers (TOT) course aimed at strengthening knowledge of key COVID-19 characteristics and control principles in healthcare stakeholders [4]. Prior disasters have highlighted that partnerships between academic and humanitarian organizations can disseminate information rapidly [4,5]. The knowledge and expertise of academic institutions and researchers combined with the ability of the humanitarian organization to scale up established partnerships to disseminate education make such partnerships ideal for efficient programming, particularly in pandemic response situations [5].

The novelty of SARS-CoV-2 and evolving knowledge of the virus emphasizes the importance of educating the healthcare workforce on how best to respond to its pandemic spread. COVID-19 disease has a wide range of clinical presentation, and the rapid increase in patients requiring healthcare due to exponential community spread of SARS-CoV-2 has resulted in a dire need for careful allocation of scarce resources and the utilization of surge capacity protocols [6,7].

This course was one of the first virtual, large scale, comprehensive training available on a wide range of key COVID-19 topics [4]. The need for virtual training has been paramount in health and medical education since the start of the pandemic, and virtual learners have reported feeling empowered and efficient amidst the uncertainties of a global emergency of this magnitude [8]. Pandemic-related issues addressed by the Project HOPE® curriculum include triage systems, inventory of critical resources, creation, and implementation of surge sites for patient care.

The contribution of this paper is to describe a novel and innovative partnership between an academic department of emergency medicine and a humanitarian nonprofit to provide a virtual training curriculum during a burgeoning pandemic, and to assess the impact of participation in the course on knowledge gains within core competencies related to COVID-19. The creation and evolution of the curriculum is described and detailed information about program implementation is presented to illustrate how similar programming could be replicated and rolled out in future humanitarian disaster scenarios requiring a rapid scale up and accessible high-quality information distribution.

## Methods

### Program development

The training program was created by a multidisciplinary team from Brown University that included faculty with specializations in infectious disease, humanitarian response, emergency care and medical education. The program includes eight focused modules on COVID-19 principles including infection prevention and control, screening and triage, diagnosis and management, stabilization and resuscitation, surge capacity, surveillance, and risk communication and community education. Whenever possible, guidance in the curriculum was taken from the best available evidence, or expert opinion via the World Health Organization (WHO) or Centers for Disease Control (CDC). Given the dynamic nature of COVID-19 clinical updates, the training program was updated by faculty members on a bi-weekly basis or earlier if needed.

### Program delivery

Project HOPE® identified participants in collaboration with local partners in each of the countries where the training was conducted. Participants had a wide range of roles including clinicians, public health workers, community health workers, policy makers, and hospital administrators. The primary training was delivered via Zoom™ over a four-day period for 3 hours each day. The content included interactive didactic modules, case-based learning, and video simulations. The videos and cases were integrated between the didactic modules. The cases included a public health scenario or patient-care scenario, and encouraged audience participation via the chat feature on Zoom™. Each video and case simulation included discussion afterwards. The course was taught in English and Spanish, or with live interpreters present for translation into other local languages. During the delivery, one instructor presented the training while another instructor was available to respond to live chat questions or comments from the audience. The trained individuals were encouraged to conduct secondary training locally using the skills and knowledge from the primary training and the entire TOT program materials were available to each participant including the teaching manuals, presentations, video simulations, practicums, feedback forms and assessment questions to aid in the secondary training to be conducted by the primary participants.

### Study design

The study used a pre and post-test design to address the following research questions:

1. What is the mean increase in knowledge of COVID-19 knowledge following targeted educational training?
2. Is the increase in COVID-19 knowledge different across the WHO countries participating in the training?
3. As a secondary analysis, was there a difference in the gain in knowledge as measured by the survey items?

Training evaluation was assessed at the end of the COVID-19 training session to gain feedback on the course.

### Program assessment and statistical analysis

To assess knowledge acquisition, a ten-question pre-test and a post-test were administered to the participants (Please see appendix 1). Participants were asked to complete the pre-test before starting the training on the first day and were asked to complete the post-test immediately following the 4th day of training. The summed correct responses of the pre and post survey were used to generate knowledge scores. Given the research design, the paired t-test was considered the most appropriate analysis to provide inference on the change in mean COVID-19 knowledge assessment scores. To examine the interaction of change by WHO geographical region, a fixed effect linear model was conducted, incorporating time and WHO geographical region The mean pretest score was the referent for the time variable, and the Southeast Asian Region was randomly used as the reference for comparison to the other WHO regions. In addition to these analyses, the change in overall percent correct by knowledge item was reported with 95% confidence interval (CI).

## Results

### Participation

**Figure 1.**
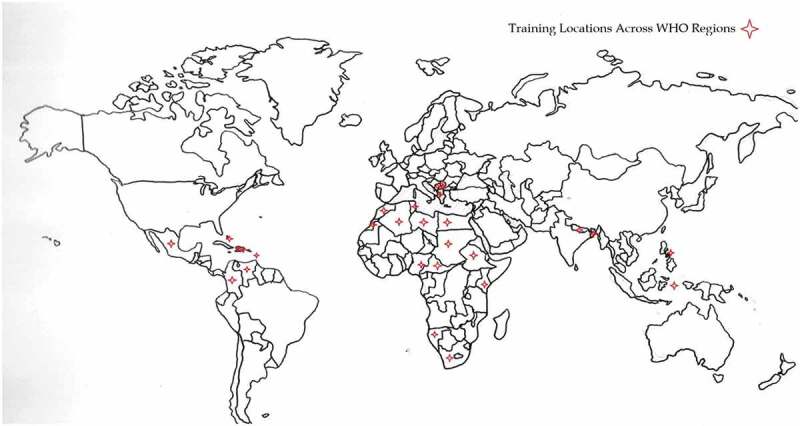
Countries where primary training was conducted.

As of January 2021, there were 4291 total participants in primary training and nearly 97,809 participants in secondary training. The pre-test was completed by 1,198 participants, representing 55 countries in all six WHO regions. Figure 1 shows countries where primary training was conducted.

### Gain in COVID-19 knowledge

The post-test was completed by 706 (58.9% of those who took the pre-test) participants. As can be seen in Table 1, the mean score on the pre-test was 6.9 (SD = 1.4, maximum = 10) and on the post-test was 8.1 (SD = 1.4). The mean change in score was 1.2 (95%CI 1.06–1.28, p = 0.01). The paired t-test supported this significant increase in knowledge scores, t(705) = 20.8, p < 0.001.

**Table 1.** Pre and post knowledge overall and by region

Trainees *n (%)	Pre Test Score - Mean (SD), Min, max	Post Test Score - Mean (SD),Min, max
All, N = 1198	6.9 (1.4), 2, 10	8.1 (1.4), 3, 10
Africa region = 327(27.3%)	7.2 (1.3), 2, 10	8.3 (1.2), 5, 10
Americas = 350(29.2%)	6.8 (1.3), 2,10	7.8 (1.4), 4, 10
Eastern Mediterranean = 85(7.1%)	7.3 (1.2), 5, 10	8.2 (1.1), 6,10
European = 127(10.6%)	6.7 (1.6), 3,10	7.6 (1.7)3, 10
South East Asia = 219 (18.3%)	6.7 (1.5), 2, 10	8.4 (1.4), 3, 10
Western Pacific = 90(7.5%)	7.3 (1.2), 4, 10	8.7 (1.4), 3, 10

*n (%) = pre training;

### WHO regional differences

**Figure 2.**
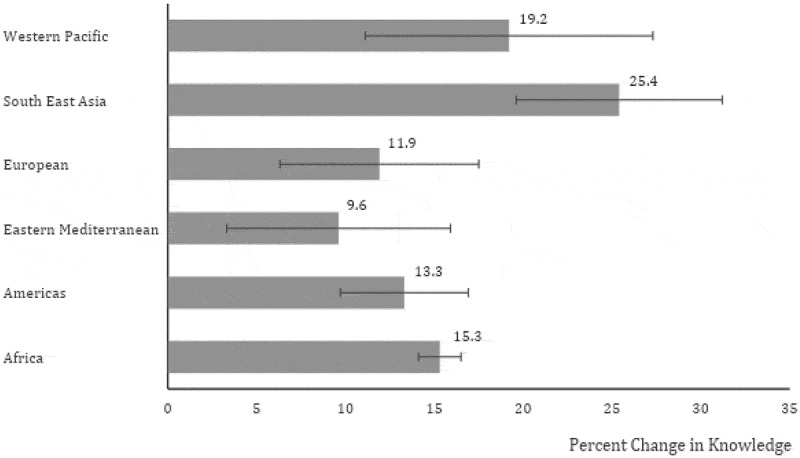
Percent change in mean pre to post knowledge score by WHO region.

After adjusting for baseline differences in knowledge, all regions significantly increased their knowledge score (p < 0.0001), but within region comparison demonstrated that comparative to the Southeast Asian region, all other regions, except the Western Pacific Region, had significantly less gain in knowledge (p = 0.001). Figure 2 shows the relative percent gain in pre to post course knowledge by each of the 6 WHO regions.

### Knowledge gain by survey items

Analysis of the change in percent correct across the 10 knowledge items was conducted. There was a significant increase in percent correct increase across all but one module topic, with a delta range from 1.4% to 44.4%. Only one module topic (evaluating breathing of patients) did not show significant percent increase in correct response (see Table S4). The three most substantial increase in change in percent correct were in the topic areas of assessing screening steps (Δ = +17%, 95% CI: 14.1, 19.9%), surge site (Δ = +21.5%, 95% CI: 17.4, 25.6%), and reporting requirements (Δ = +44.4%, 95% CI: 37.8, 49%). (See Table 2)

**Table 2.** Item analysis of knowledge change

Module Topic	Pre-Frequency of Correct – n, % (95% CI)	Post-Frequency of Correct – n, % (95% CI)	Change in Frequency of Correct – % (95% CI)
Shared traits SARS-CoV-1 and SARS-CoV-2	1083, 9.1.9% (±1.6%)	683, 96.7% (±1.3%)	4.8% (± 1.6%)
Precautions	1008, 85.5% (±2.2%)	635, 89.9% (±2.3%)	4.4% (± 1.6%)
Evaluating breathing	1066, 90.4% (±1.8%)	637, 90.2% (±2.3%)	−0.2% (± 0.3%)
Complications	913, 77.4% (±2.7%)	643, 91.1% (±2.2%)	13.7% (± 2.7%)
Authorities reporting requirements	219, 18.6%(±5.2%)	445, 63% (±4.5%)	44.4% (± 4.6%)
Three screening steps	883, 74.9%(±2.9%)	649, 91.9% (±2.1%)	17.0% (± 2.9%)
Reduce potential spread	1144, 97% (±1.0%)	695, 98.4% (±1.0%)	1.4% (± 1.5%)
Surge site	406, 34.4% (±4.6%)	395, 55.9% (±4.9%)	21.5% (± 4.1%)
Communication and trust	412, 34.9% (±4.6%)	296, 41.9% (±5.6%)	17.0% (± 4.3%)
Social stigma	1066, 90.4% (±1.8%)	669, 94.8% (±1.7%)	4.4% (± 1.6%)

### Training evaluation

In the qualitative data from participant feedback, 92% of respondents (n = 623) reported that the training provided to them was at the right level for their training. 31.8% of respondents had no specific feedback and reported the training was ‘Good’ and 16.2% asked for more video/exercises/cases.

## Discussion

SARS-CoV-2, like previous global health emergencies, has highlighted how partnerships between academic institutions and humanitarian organizations can develop substantive public health responses [4,9]. The current report and data show that this intersectoral collaboration has helped strengthen knowledge dissemination during the COVID-19 pandemic by leveraging the strengths of each partner. All items assessed showed a significant increase in knowledge gain except one item on breathing evaluation. A potential reason for this is that oxygen cutoffs and their management for stable patients vary across different regions, and non-clinician participants could have difficulty understanding the different responses.

The partnership capitalized on the international network of established partners for Project HOPE® and the scientific and educational expertise of Brown University to lead to a rapid and effective dissemination of education material regarding COVID-19 around the world. The success of this partnership is reflected in the numbers of health care providers trained and the significant improvement in knowledge immediately after taking the course. The virtual format of this training has several advantages. First, it is a low-cost alternative to in-person training. A 2019 study showed that the average travel cost for an in-person conference is $177 US dollars for domestic travel and $1012 for international travel [10]. In addition to travel costs, conference participants must also consider the costs of accommodations and meals, as well as possible loss of pay or use of vacation time to allow for travel to a conference. This ultimately may render an in-person course cost-prohibitive. Additionally, the virtual format allows for social distancing so that participants can safely train while minimizing COVID exposure risk. It also provides an alternative for those who choose to decrease their carbon footprint [10].

Furthermore, this platform has allowed for videos, simulations, and lectures to be easily accessible by participants across the globe. As the COVID-19 pandemic continues to evolve and new therapeutics and vaccines develop, academic humanitarian partnerships such as the one described here represent an impactful and efficient means for disseminating evolving knowledge. To meet the demand for ongoing education needs, this partnership has now been conducting vaccine-related training as well.

Prior studies have shown the success in knowledge improvement of virtual training programs, and our study shows knowledge gain for a larger scale training on a topic with evolving knowledge concepts [11,12]. Our results are encouraging for future educators to replicate such programs during pandemic settings. One major aftermath of the pandemic is the rise in mental health conditions in a world where education and access to mental health problems are scarce [13,14]. Programs such as this could be replicated for topics of mental health awareness and education of healthcare workers globally as well. Furthermore, it has been well documented that the best way to lower vaccine hesitancy and misinformation is through healthcare worker’s communication, and these trainings provided a platform for direct communication by health care providers, in primary and secondary training [15].

## Limitations

This study has several limitations. First, primary trainings were only conducted in English or Spanish, potentially creating a language barrier to the understanding of the materials for non-primary English or Spanish-speaking participants. However, secondary cascade trainings were conducted in a variety of local languages for local health workers by participants who completed the primary training. An issue that arose due to digital literacy with the Zoom™ platform was the unintentional unmuting by participants. This was overcome by muting all participants to begin the training and having a second instructor monitor the chat and to mute any unmuted participants. Additionally, though pre- and post-test participation was encouraged, a large proportion of trainees did not complete either the initial or the final assessments. Only 60% of participants who completed the pre-test also completed the post-test, limiting our ability to fully evaluate the change in test scores as a proxy for knowledge achievement. Our work did not show any clear trends among who did or did not respond and while there could be potential response bias, there is no clear explanation for our moderate follow through response rate. One could conjecture that because volunteers were not compensated for the participation in the post-test, after the conclusion of the didactic, they did not have the same motivation as participants did in the pre-test. Fatigue from the length of training course could have also played a role in lower response rate in post-test compared to pre-test. Making the pre- and post-test mandatory or providing incentive to complete could have likely increased the response rate. Although we show improved knowledge in the short term, our study timeline, and resources were not able to assess whether this changed behavior or practice pattern for trainees, or if there were downstream improvements in clinical care. The clinical impact on patient care or acquisition of practical skills remains unknown.

## Conclusion

This study shows that a large-scale virtual TOT program led to increase in knowledge gain in COVID-19 concepts across all WHO regions. Academic–humanitarian partnership, such as described here for the COVID-19 pandemic, can serve as a valuable instrument to both create and disseminate educational programs rapidly across the globe. Subsequently, tens of thousands of individuals were further trained in local secondary training around the globe. This model is an economical and effective way to educate individuals globally, especially in the face of pandemic travel restrictions, and can serve as both an example and precedent for future efforts rapidly responding to a number of potential infectious diseases, ecological disasters, or other challenges that strain local and international health systems. Future work could look at long-term knowledge retention and behavior changes as a result of such trainings.

## Paper context

Evolving knowledge and limited training of frontline workers is a barrier to pandemic response. To address this gap during COVID-19, an innovative, virtual, training of trainers program was designed in collaboration between an academic and humanitarian organization. This paper presents findings from this training, with was effectively disseminated to all WHO regions with nearly 100,000 primary and secondary participants trained. Early innovative partnerships like this can play an important role in global pandemic response.
